# Western diet associated with increased post-stroke depressive symptoms

**DOI:** 10.1017/jns.2022.38

**Published:** 2022-06-09

**Authors:** Laurel Cherian, Puja Agarwal, Thomas Holland, Julie Schneider, Neelum Aggarwal

**Affiliations:** 1 Rush Department of Neurological Sciences, Rush University Medical Center, 1725 W Harrison St, Chicago, IL 60612, USA; 2 Rush Alzheimer's Disease Center, 1750 W Harrison St, Chicago, IL 60612, USA; 3 Rush Institute for Healthy Aging, 1653 W. Congress Parkway, Chicago, IL 606012, USA

**Keywords:** Aging, Depression, Stroke, Western diet

## Abstract

The present study examines the association of diet with depressive symptoms among stroke survivors from a community cohort of older adults. Depression is common after stroke. A healthy diet has previously been associated with fewer depressive symptoms in older individuals, but it is unknown if this effect is also seen in stroke survivors. Eighty-six participants from the Memory and Aging Project with a history of stroke at their study baseline enrolment, complete dietary data and two or more assessments for depression were included in this observational prospective cohort analysis. Depressive symptoms were assessed annually with a 10-item version of the Center for Epidemiologic Studies Depression scale. Diet was assessed using a validated food-frequency questionnaire administered at baseline. Diet scores were based on analysis of participants’ reported intakes of 144 food items. A generalised estimating equation (GEE) model was applied to examine the association of diet score with depressive symptoms. The study participants had a mean age of 82 ± 7⋅17 years and 14⋅42 ± 2⋅61 years of education, and 82⋅56 % were female. Western diet score was positively associated with depressive symptoms over time (diet score tertile 3 *v*. tertile 1: *β* = 0⋅22, se = 0⋅09, *P* = 0⋅02; *P* for trend = 0⋅022). Interaction with sex suggested a stronger effect in females. A Western diet was associated with more post-stroke depressive symptoms, suggesting nutrition is important not only for reducing cerebrovascular risk, but for protecting post-stoke mental health as well.

## Introduction

Depressive symptoms are common after stroke, affecting up to 30 % of patients^(1)^, compared with 13 % of population controls^(2–4)^. These symptoms may continue to linger long-term, with some studies showing as many as 25 % of post-stroke patients continuing to experience depressive symptoms 5 years post-stroke^(1)^. Post-stroke depression has been associated with worse outcomes, lower likelihood of returning to pre-stroke activities and higher risk of recurrent stroke^(2)^. In addition, post-stroke depression has been associated with increased all-cause mortality in stroke survivors^(5)^.

Treatment with antidepressant medications and behavioural therapy has been shown to effectively reduce the burden of depressive symptoms in stroke patients but may not completely alleviate them. Antidepressants are occasionally associated with undesirable side effects, making compliance or titration to effective doses challenging^(6)^.

Complementary approaches to reduce the burden of post-stroke depressive symptoms are needed. A healthy dietary pattern has been associated with a reduced risk for depressive symptoms in a variety of studies, but there are relatively few studies examining the role of diet on depressive symptoms in stroke survivors. Therefore, the present study sought to examine the association of various diet patterns on the prevalence of depressive symptoms in a community cohort of older adults who are stroke survivors.

## Methods

### Study population

The present study was conducted using data from the Rush Memory and Aging Project (MAP), an ongoing study of volunteers living in retirement communities and senior public housing units in Chicago, Illinois. The ongoing open cohort study began in 1997 and includes annual clinical neurological examinations, as previously described^(7)^. Beginning in 2004, MAP study participants began to complete comprehensive food-frequency questionnaires (FFQs). Of the 2178 older persons enrolled in the MAP study, 1412 had complete dietary data that could serve as the baseline. Of these, 945 also had two or more annual screens for depressive symptoms. Of these, 86 participants had a clinical history of stroke. These 86 MAP participants, who had complete comprehensive FFQs, at least two annual screens for depressive symptoms, and a history of stroke were included in the analysis. The average study follow-up time since first dietary assessment was 6⋅6 years. This study was conducted according to the guidelines laid down in the Declaration of Helsinki and all procedures involving human subjects/patients were approved by the Institutional Review Board of Rush University Medical Center. Written informed consent was obtained from all subjects/patients.

### Depression evaluations

Participants completed a 10-item version of the Center for Epidemiologic Studies Depression (CESD) scale at each evaluation^(8)^. Individuals were asked if they had experienced any of the 10 depressive symptoms in the past week (e.g. ‘I felt like everything I did was an effort.’). The score is the total number of symptoms experienced, with a range of 0–10. A high burden of depressive symptoms was defined as a score greater than or equal to 4^(9)^. The use of antidepressant medication was also recorded.

### Dietary pattern scoring

Dietary pattern scores were based on responses to a modified Harvard semi-quantitative FFQ that was validated for use in older Chicago community residents^(10)^. The typical frequency of intake of 144 food items over the prior 12 months was reported by participants. The caloric content and nutrient levels for each food item were based on age- and sex-specific portion sizes according to national dietary surveys or logical portion size (e.g. a slice of bread). Details of the dietary components and maximum scores for the MIND, DASH and Mediterranean diets have been previously reported^(11–13)^.

Briefly, the MIND diet score is based on the consumption of ten healthy food groups (leafy green vegetables, other vegetables, nuts, berries, beans, whole grains, fish, poultry, olive oil and wine) and five unhealthy food groups (red meats, butter and stick margarine, cheese, pastries and sweets, fried food and fast food). If olive oil was reported as the primary oil used at home, it was scored 1; otherwise, it was scored 0. For the remaining components, the frequency of consumption of the food items corresponding with each component was summed and given a concordance score of 0, 0⋅5 or 1, with 1 representing the highest concordance^(11)^. The final MIND diet score is the sum of the fifteen component scores.

The DASH diet is based on the consumption of three dietary components (total fat, saturated fat and sodium) and seven food groups (grains, fruits, vegetables, nuts, seeds and legumes, dairy and meat)^(13)^. Scores of 0, 0⋅5 and 1 were assigned to each food group based on the frequency of consumption. Total possible scores ranged from 0 (lowest) to 10 (highest) diet concordance.

The Mediterranean dietary pattern was based on the MedDiet score as described by Panagiotakos and colleagues^(12)^ that uses the serving quantities of the traditional Greek Mediterranean diet as the comparison metric. Eleven dietary components (non-refined cereals, potatoes, fruits, vegetables, legumes, fish, red meat and products, poultry, full-fat dairy products, use of olive oil in cooking and alcohol) are each scored from 0 to 5 and then summed for a total score ranging from 0 to 55 (highest concordance).

The Western dietary pattern was derived using principal component analysis using the forty foods or food groups described by Hu and colleagues^(14)^. The eigenvalue of more than 1 was considered, and based on the food group consumption in the study population our analysis retained two factors with distinct food groups (factor loading >0⋅20). We labelled factor 1 as healthy diet and factor 2 as Western diet and then computed the factor score for each pattern by combining the selected food groups observed for that pattern with weights proportional to their individual components and factor loadings^(15)^. The Western dietary pattern was characterised by the intake of food items that are processed and/or high in fat and simple carbohydrates, including red and processed meat, eggs, refined grains, French fries, high-fat dairy products, sweets and snacks (Supplementary Table S2).

### Covariates

Non-dietary variables were obtained at participants’ baseline clinical evaluations through a combination of clinical evaluation, self-report, medication inspection and measurements. The process is identical to that performed in the Religious Orders Study and was designed to reduce costs and enhance uniformity of diagnostic decisions over time and space^(16)^. Participants self-reported their birth date and years of education. Physical activity was determined by participants’ self-reported minutes spent over the previous 2 weeks on five activities (walking for exercise, yard work, calisthenics, biking and water exercise)^(17)^. A modified 10-item version of the Center for Epidemiological Studies Depression (CESD) scale was used to evaluate depressive symptoms, with responses categorised into a binary outcome with greater than or equal to four depressive symptoms versus fewer than four depressive symptoms^(8)^. Hypertension was defined by the average of two blood pressure measurements, ≥160 mmHg (systolic) or ≥90 mmHg (diastolic), or a reported clinical history of hypertension or current use of antihypertensive medications. Myocardial infarction history was based on the current use of cardiac glycosides (e.g. lanoxin or digoxin) or self-reported history. Clinical history of diabetes was obtained by self-reported medical diagnosis or current use of diabetic medications. Diagnosis of stroke was obtained through a combination of clinical evaluation and self-report to the question ‘Has a doctor, nurse, or therapist ever told you that you have had a stroke?’^(18)^. Medication use was based on interviewer inspection.

### Statistical analysis

The data were summarised using median and quartiles, mean and sd, or number (relative frequency), as appropriate. Separate generalised estimating equation (GEE) models were applied for the longitudinal analysis of the burden of depressive symptoms as a binary outcome, defined as greater than or equal to four depressive symptoms *v*. fewer than four depressive symptoms, of the dietary exposures (MIND, DASH, Mediterranean and Western diets scores) in order to describe the relationship between dietary patterns and depressive symptoms over time in older adults with a history of stroke. The four dietary patterns were examined in two separate models, an age-adjusted model and a basic-adjusted model that included the following potential confounders previously associated with depression: age, caloric intake, education, sex and use of antidepressant medications. Additional adjustments were then made for cardiovascular disease (hypertension, diabetes and myocardial infarction). The dietary scores were modelled as indicators of the top two tertiles in each of these models. A test of linear trend was applied to each by assigning the median tertile intake level to all those in a given tertile and modelling as a single categorical variable. We further investigated the interaction of dietary pattern with age, sex and education on depressive symptoms.

## Results

The study participants had a mean age of 82 ± 7⋅17 years and 14⋅42 ± 2⋅61 years of education, and 82⋅56 % were female. Baseline characteristics of our analytical sample overall and as per tertile of Western diet score are presented in Table 1. The median overall diet scores for the population were as follows: DASH 4⋅00 (IQR 3⋅00, 5⋅00), Mediterranean 32⋅00 (IQR 28⋅00, 35⋅00), MIND 8⋅00 (IQR 7⋅00, 9⋅50) and Western 3⋅41 (IQR 2⋅24, 4⋅41). Our analytical sample was slightly older (82 ± 7⋅17 *v*. 81 ± 7⋅31) and included more females (85 % *v*. 74 %) compared to the overall MAP cohort (Supplementary Table S2). The mean Western diet score in study participants with baseline stroke was 3⋅41, comparable to the score of 3⋅52 for overall MAP participants without any stroke at baseline.

**Table 1.** Baseline characteristics of the 86 study participants of the memory and aging project by tertile of Western diet score

	Overall	Tertile of Western diet score		
		T1	T2	T3
*N*	86	29	28	29
Western diet score, median (IQR)	3⋅10 (2⋅24, 4⋅41)	1⋅98 (1⋅44, 2⋅24)	3⋅10 (2⋅79,3⋅54)	4⋅91 (4⋅41,6⋅04)
Age, mean ± sd, years	82 ± 7⋅17	79⋅8 ± 7⋅8	83⋅4 ± 7⋅2	83⋅5 ± 6⋅1
Male, %	15 %	10 %	18 %	24 %
Education, mean ± sd, years	14⋅4 ± 2⋅6	14⋅4 ± 2⋅5	13⋅9 ± 2⋅2	15⋅0 ± 3⋅0
Total energy, mean ± sd, kcal/d	1827 ± 525	1398 ± 414	1852 ± 328	2231 ± 448
Diabetes, %	17 %	10 %	18 %	31 %
Hypertension, %	68 %	72 %	75 %	90 %
Myocardial infarctions, %	27 %	31 %	32 %	31 %

IQR, interquartile range; sd, standard deviation; BMI, body mass index.

We investigated the relation of different dietary patterns with depressive symptoms in those with a history of stroke, adjusting for confounding variables including age, sex, education, caloric intake and use of antidepressant medications. Those with the highest Western diet scores were positively associated with developing depressive symptoms over time, as compared to those with the lowest scores (diet score tertile 3 *v*. tertile 1: *β* = 0⋅23, se = 0⋅10, *P* = 0⋅02; *P* for trend = 0⋅022; Table 2). There were no significant differences between tertiles for the Mediterranean (−0⋅0542, se 0⋅10, *P* = 0⋅59), DASH (−0⋅23, se 0⋅15, *P* = 0⋅13) and MIND (−0⋅11, se 0⋅11, *P* = 0⋅34) diets. When additionally adjusted for cardiovascular conditions, including hypertension, diabetes, and myocardial infarction, the Western diet association with depressive symptoms was retained (Table 2).

**Table 2.** Estimated effects (*β* coefficient^a^ (se, *P*-value)) of Western diet on depressive symptoms (CESD >/= 4) among stroke survivors in Memory and Aging Project over the average follow-up of 6⋅6 years

	*N*	Tertile 1	Tertile 2	Tertile 3	*P* for trend
			*β* coefficient^a^	*β* coefficient^a^	
			(se, *P*-value)	(se, *P*-value)	
Wester Diet association, overall					
Basic model		Ref	0⋅114	0⋅229	0⋅03
			(0⋅09, 0⋅209)	(0⋅10, 0⋅024)	
Basic + CVD^b^		Ref	0⋅111	0⋅223	0⋅02
			(0⋅10,0⋅250)	(0⋅09, 0⋅019)	

GEE models. Basic model adjusted for age, sex, education, calories and use of antidepressant medication.

a *β* coefficient from the model for the interaction term between Western diet score and time.

b CVD, cardiovascular conditions included diabetes, hypertension and myocardial infarction.

We further investigated whether the observed association between the Western diet and depressive symptoms was modified by age, sex and education. The only statistically significant modification of effects was that for Western diet by sex (*P* < 0⋅001). The interaction with sex suggested a stronger effect in females.

All healthy diet patterns (MIND, Mediterranean and DASH) were positively correlated with each other (0⋅68 < Rho < 0⋅74, *P* < 0⋅0001) and the Western diet score was negatively correlated with the MIND and DASH diet scores (Rho = −0⋅29, *P* = 0⋅0074; Rho = −0⋅041, *P* = 0⋅0001). The Western and Mediterranean diet scores were not correlated (Rho = 0⋅07, *P* = 0⋅53).

## Discussion

Post-stroke depressive symptoms are common and disabling. Our prospective study among older adults with a history of stroke found that a Western dietary pattern was associated with higher rates of depressive symptoms during the follow-up period, contributing towards a better understanding of the links between nutrition, stroke and depression.

Prior studies examining a traditional Western diet, containing high levels of refined sugars and carbohydrates, fried foods and saturated fats, have also found associations with higher rates of depression. We previously found an association between a healthy dietary pattern and fewer depressive symptoms in all MAP cohort study participants^(19)^ as well as slower cognitive decline in MAP stroke survivors with a healthy dietary pattern^(20)^. The present study extends our previous results, again suggesting that diet may be important in combating depression, both in the general population and in stroke survivors. Although the sample size of this analysis was small and may account for the lack of significant relations with other healthy diet scores, such as the MIND, DASH or Mediterranean diets, the strongly positive association between the Western diet and more depressive symptoms over time suggests the continued to need to examine these findings in other cohorts and the ongoing trials.

Researchers from the Whitehall II longitudinal study found a detrimental influence of the Western diet on depressive symptoms in middle-aged women^(21)^. A separate study from the Whitehall II prospective cohort found that in middle-aged adults, increased intake of ‘processed food’ such as sweetened desserts, fried food, processed meat, refined grains and high-fat dairy products was associated with increased odds of depression over time based on the CESD score^(22)^. The Western diet was also associated with higher General Health Questionnaire scores (ranging from 0 to 12, with a higher score indicating poorer mental health) in a longitudinal, age-stratified, randomly selected population-based sample of 1494 women in the Geelong Osteoporosis Study in Australia^(23)^.

While psychosocial factors, such as adjusting to a new disability or the loss of independence, are certainly important contributors to post-stroke depression, there is also evidence that depression may be rooted in neurochemical abnormalities consistent with a pro-inflammatory state, including elevations in C-reactive protein and cytokines such as interleukin 6 and tumour necrosis factor alpha (TNF-α)^(24)^. Antidepressant medications have been shown to decrease these pro-inflammatory cytokines^(25)^, further linking inflammation and depression.

An increase in pro-inflammatory cytokines also occurs at the onset of a stroke, when an innate immune response is triggered, resulting in brain inflammation^(25)^. Elevated levels of IL-1, TNF-α and IL-6 have been observed post-stroke in brain regions associated with mood regulation, including the hippocampus and striatum^(26)^. Delayed induction of anti-inflammatory IL-10 is seen post-stroke as a compensatory response, peaking within the first week. Also, the brain-derived neurotrophic factor (BDNF) is a widely expressed neurotrophin, particularly in the hippocampus and cortex. Low BDNF levels have been associated with post-stroke depression, with one study correlating low levels at the time of admission for acute stroke with rates of post-stroke depression at 3 months^(27)^. BDNF is involved in the maturation of axons and dendrites, neurotransmitter release and regulation of long-term potentiation.

Relatively little data are available on inflammatory biomarkers in relation to depression in the chronic post-stroke phase. Given that post-stroke depression may linger for years, it may be useful to examine in the long term whether pro-inflammatory biomarkers vary between individuals with a higher burden of chronic post-stroke depressive symptoms and those who are less affected.

It is currently unclear how nutrition affects post-stroke depression on a cellular level, but a healthy dietary pattern may shift the body to an anti-inflammatory state, helping to combat the oxidative stress induced by stroke that has been hypothesised to contribute to post-stroke depression^(28)^. Reactive oxygen species, which are created during stroke, lead to oxidative stress, lipid peroxidation, protein oxidation and DNA damage in neural tissues. The presence of antioxidant nutrients and bioactive substances found in a healthy dietary pattern, such as one high in vegetables, fruits, nuts and whole grains, may help neutralise these reactive species^(28)^.

Pilot data from our institution's inpatient stroke service, which included FFQs to obtain dietary practice during the year prior to stroke showed that patients with stroke consumed more unhealthy diet components (processed and red meats, fried food, pastries and sweets) and fewer healthy components (vegetables, whole grains, nuts, beans and legumes) than what is recommended in the MIND diet^(29)^, suggesting an opportunity for substantial improvement with dietary intervention. This, in conjunction with the findings of our present study, has helped to identify a potential treatment gap for stroke survivors suffering from depressive symptoms. While antidepressant medications improve symptoms for many stroke survivors, a comprehensive approach that includes dietary modification, in addition to pharmacologic treatment and therapy, may be the most effective way to prevent and treat post-stroke depressive symptoms.

The association between diet pattern and depressive symptoms has been explored in prior studies^(30)^, but high-risk individuals, such as stroke survivors, were frequently excluded from these community-based cohorts. Additionally, stroke is often pooled with a variety of other cardiovascular diseases, such as myocardial infarction and heart failure, making the implications for post-stroke individuals more difficult to ascertain. The present study provides novel information on the role of diet pattern on depressive symptoms in an entirely post-stroke population.

The implementation of dietary approaches can be challenging in any population and may be even more difficult in the stroke population, given what we have seen from our inpatient sample showing poor baseline dietary habits. However, to presume that a diet intervention would be futile is a missed opportunity for combating both the etiologic causes of stroke (hyperlipidaemia, hypertension and diabetes) as well as post-stroke depressive symptoms. The present study provides evidence of a potential additional mental health benefit to patients who adopt a healthier diet pattern, which may also serve as a motivator for change. Nonetheless, any future dietary approach that attempts to address this gap in post-stroke care will need to have a focus on achieving long-term adherence.

There are several limitations to the present study, with the most important being the observational nature, thus precluding the establishment of a cause-and-effect relationship, and the lack of radiographic confirmation of the clinical and/or self-reported history of stroke from this community-based cohort. Also, the present study did not find an association between the other dietary patterns, such as the Mediterranean, DASH or MIND diet and depression over time, although it is possible that such an association could appear with a larger sample size. The MAP cohort is an older, predominantly non-Hispanic white population, so findings should not be generalised to other ethnic groups or younger cohorts, although previous studies have found associations between a healthy dietary pattern and reduced depressive symptoms in these populations^(31–35)^. Also, dietary questionnaires had limited questions regarding some of the dietary components and frequency of consumption. For example, the consumption of nuts, berries, beans and olive oil were characterised by a single item each.

The strengths of the study include its use of a validated food questionnaire for comprehensive dietary assessment, multiple measures of depressive symptoms with a validated scale (CESD) over time and statistical control of the important confounding factors including use of antidepressant medications.

Depressive symptoms are a frequent complication after stroke, and they can affect functional outcomes, cognition and risk of recurrent stroke or death^(27)^. In addition to behavioural and pharmacological interventions, the adoption of a healthy diet may help reduce depressive symptoms post-stroke. Additional studies are warranted to determine the most effective approach to help stroke survivors adhere to a healthy diet and to better characterise the biomarkers, structural changes and clinical outcomes associated with dietary interventions for post-stroke depression.

## Supplementary material

For supplementary material accompanying this paper visit https://doi.org/10.1017/jns.2022.38. click here to view supplementary material
