# Supplementary material for: Western diet associated with increased post-stroke depressive symptoms
Source: J Nutr Sci. 2022 Jun 9;11:e44. doi: 10.1017/jns.2022.38 (PMC9201874; doi:10.1017/jns.2022.38)
Supplement: Supplementary file 1 [file S2048679022000386sup001.zip › Supplemental_Table_2.docx]

**Supplementary Table 2: Factor Loadings of the two significant factors - Healthy diet pattern and Western diet pattern identified using principal component analysis**

| **Rotated Factor Pattern** | | | | |
| --- | --- | --- | --- | --- |
|  | **Factor1(Prudent)** |  | **Factor2(Western)** |  |
| **Processed meat** | -10 |  | 51 | * |
| **Red meat** | 14 |  | 49 | * |
| **Organ meat** | -11 |  | 5 |  |
| **Seafood** | 44 | * | -15 |  |
| **Poultry** | 43 | * | 14 |  |
| **Egg** | 5 |  | 38 | * |
| **Butter** | -8 |  | 32 | * |
| **Margarine** | -5 |  | 33 | * |
| **Low-fat dairy** | 35 | * | -4 |  |
| **High-fat dairy** | 10 |  | 44 | * |
| **Liquor** | -8 |  | 5 |  |
| **Wine** | 12 |  | 7 |  |
| **Beer** | -5 |  | 15 |  |
| **Tea** | 23 | * | 10 |  |
| **Coffee** | -1 |  | 12 |  |
| **Fruit** | 63 | * | -3 |  |
| **Fruit juice** | 6 |  | 6 |  |
| **Cruciferous vegetables** | 63 | * | -13 |  |
| **Dark vegetables** | 68 | * | -12 |  |
| **Tomato** | 50 | * | 20 | * |
| **Green leafy vegetables** | 64 | * | -6 |  |
| **Legumes** | 57 | * | -6 |  |
| **Other vegetables** | 71 | * | -8 |  |
| **Potato** | 8 |  | 26 | * |
| **Fried food** | -17 |  | 39 | * |
| **Whole grain** | 34 | * | 4 |  |
| **Cold cereal** | 19 |  | -14 |  |
| **Refined grain** | 15 |  | 68 | * |
| **Pizza** | 5 |  | 41 | * |
| **Snacks** | 18 |  | 47 | * |
| **Nuts** | 35 | * | 34 | * |
| **High energy drink** | -15 |  | 43 | * |
| **Oil dressing** | 45 | * | 6 |  |
| **Creamy dressing** | 5 |  | 33 | * |
| **Soup** | 10 |  | 6 |  |
| **Other soup** | 31 | * | 16 |  |
| **Sweets** | -9 |  | 47 | * |
| **Condiments** | 0 |  | 42 | * |
| **Printed values are multiplied by 100 and rounded to the nearest integer. Values greater than 0.2 are flagged by an '*'.** | | | | |
