# Supplementary material for: Western diet associated with increased post-stroke depressive symptoms
Source: J Nutr Sci. 2022 Jun 9;11:e44. doi: 10.1017/jns.2022.38 (PMC9201874; doi:10.1017/jns.2022.38)
Supplement: Supplementary file 1 [file S2048679022000386sup001.zip › Supplementary_Table_1.docx]

**Supplementary Table 1: Baseline characteristics of Analytical sample of individuals with clinical history of stroke (n=86) and overall cohort with dietary data (n=1386) Memory and Aging Project**

| Variable | Analytical Sample  (N=86) | Overall Cohort (N=1386) Minus those with a history of stroke (N=86) =  (N=1300) |
| --- | --- | --- |
| Age, y, mean ± SD, years | 82 ± 7.17 | 81.20, 7.31 |
| Male, % | 15 | 25.54 |
| Education, y, mean | 14.42, 2.61 | 15.12, 3.01 |
| Total energy mean ± SD , kcal/day | 1826.70, 524.67 | 1772.57, 562.49 |
| Western diet score, mean | 3.41 | 3.52 |
| MIND diet score, mean | 7.98 | 7.92 |
| MedDiet score, mean | 30.83 | 31.28 |
| DASH score, mean | 4.02 | 3.91 |
